# Protonic Capacitor: Elucidating the biological significance of mitochondrial cristae formation

**DOI:** 10.1038/s41598-020-66203-6

**Published:** 2020-06-29

**Authors:** James Weifu Lee

**Affiliations:** Department of Chemistry and Biochemistry, Old Dominion University, Norfolk, VA 23529 USA

**Keywords:** Biochemistry, Biophysics, Cell biology, Chemistry, Energy science and technology, Physics

## Abstract

For decades, it was not entirely clear why mitochondria develop cristae? The work employing the transmembrane-electrostatic proton localization theory reported here has now provided a clear answer to this fundamental question. Surprisingly, the transmembrane-electrostatically localized proton concentration at a curved mitochondrial crista tip can be significantly higher than that at the relatively flat membrane plane regions where the proton-pumping respiratory supercomplexes are situated. The biological significance for mitochondrial cristae has now, for the first time, been elucidated at a protonic bioenergetics level: 1) The formation of cristae creates more mitochondrial inner membrane surface area and thus more protonic capacitance for transmembrane-electrostatically localized proton energy storage; and 2) The geometric effect of a mitochondrial crista enhances the transmembrane-electrostatically localized proton density to the crista tip where the ATP synthase can readily utilize the localized proton density to drive ATP synthesis.

## Introduction

**Figure 1:**
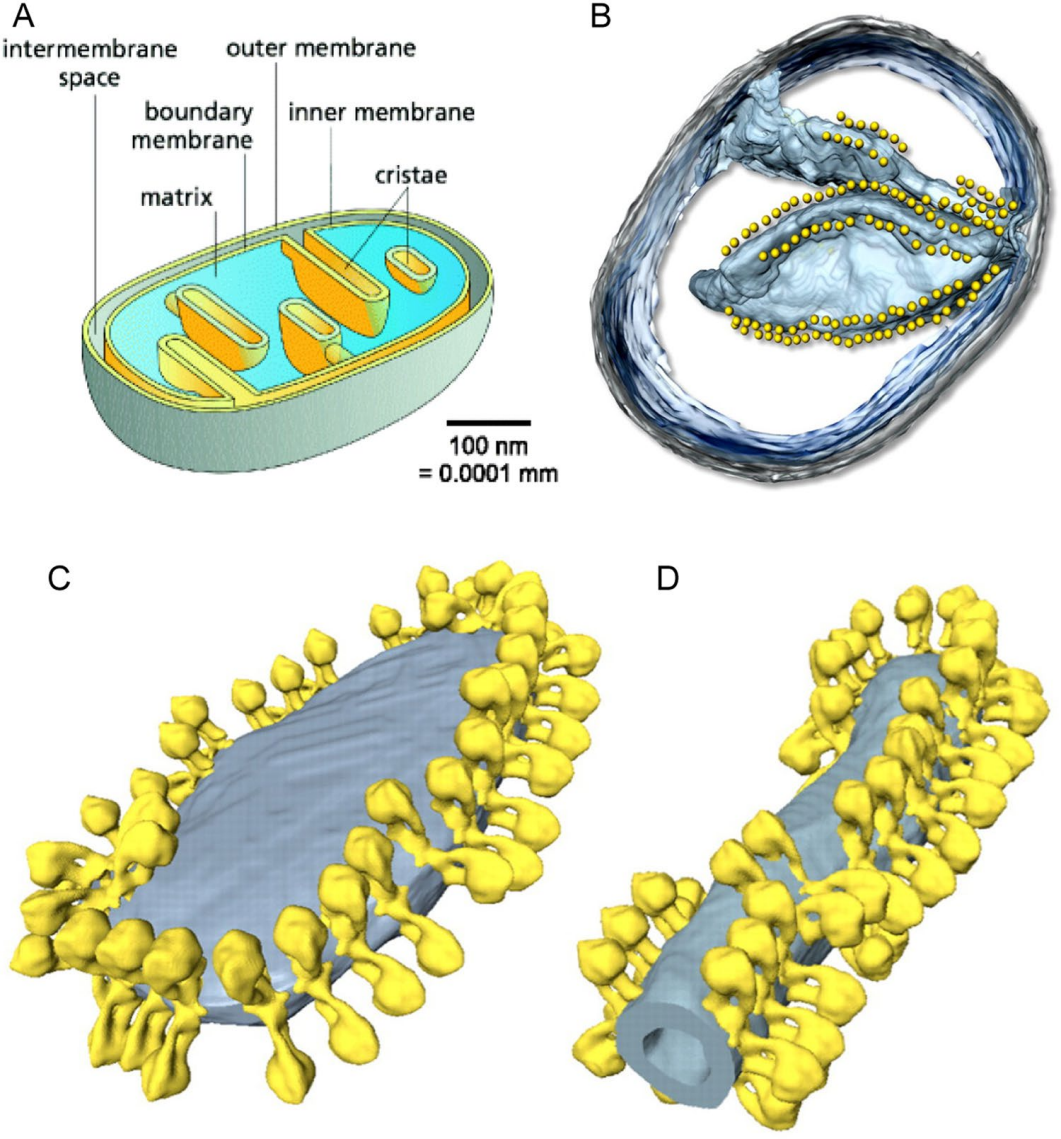
(**A**): Illustration of a mitochondrion with cristae (adapted/modified from Fig. 1 of Kuhlbrandt *et al*.^10^). The mitochondrial outer membrane surrounds the inner membrane which separates the inter-membrane space from the matrix. The inner membrane is differentiated into the inner boundary membrane and the cristae. The two regions of the inner membrane are continuous at the crista junctions. The cristae extend more or less deeply into the matrix. (**B**): Segmented surface of a mitochondrion showing position of ATP synthase dimers (yellow spheres) to cristae membrane (blue) as revealed by electron cryotomography (adapted from Fig. 1 of Davies *et al*.^11^). (**C** and **D**): ATP synthase distribution in mitochondrial cristae showing rows of ATP synthase dimers along the highly curved ridges of disk-shaped (**C**) or tubular (**D**) cristae vesicles as observed through electron cryotomography (adapted from Fig. 4 of Davies *et al*.^12^).

Why mitochondria develop cristae? Mitochondria are the major powerhouses in nearly all cells of the eukaryotic life such as animals including human. Although mitochondria and their associated biomolecules and bioenergetics^1–8^ have been studied for more than 50 years, they still remain today as an intriguing research topic of fascinating and unexpected new insights^9^. As illustrated in Fig. 1, mitochondrial cristae are disk-like lamellar invaginations that extend from the inner boundary membrane into the matrix space, and they are continuously connected with the inner boundary membrane through the crista junctions within a mitochondrion^10–13^. An evolutionarily conserved “mitochondrial contact site and cristae organizing system” is also believed to associate with the formation and stabilization of crista junction structures that connects cristae with the boundary inner membrane^14^. A key factor in shaping cristae membranes is the formation of dimeric and/or oligomeric F_1_F_o_-ATP synthase complexes at the tips/rims (curved ridges) of cristae (Fig. 1C,D)^10,13,15–17^. It has been reported that the human mitochondrial F_1_F_o_-ATP-synthase dimers are formed by interlinking each pair of their monomers with a protein linker, resulting in long arrays of the dimers along the curved ridges of the cristae^18^.

Conversely, it is now quite clear that the proton-pumping “respiratory supercomplexes”^19–22^ which comprise the redox-driven proton-pumping complexes I, III and IV are situated primarily at the relatively flat membrane plane regions of mitochondrial cristae whereas the mitochondrial ATP synthase dimers are distributed at the curved cristae tips and ridges^10–13,20,21^. This amazing feature has been well observed in both intact mitochondrial samples^10–12,23^ and reconstitution experiments^13^ using cryo-EM and electron cryo-tomography (cryo-ET) in combination with X-ray crystallography^24,25^. In the past, mitochondrial cristae membrane “was hypothesized to serve as a specialized compartment ensuring optimal conditions for ATP production” based on empirical understanding^1,26^, but not at the protonic bioenergetics level. So far, it is still rather puzzling as to the questions of: 1) what is the biological significance for the formation of cristae in mitochondria in relation to proton-coupling bioenergetics and 2) why the F_1_F_o_-ATP-synthase dimers are distributed primarily at the curved ridges of mitochondrial cristae whereas the proton-pumping “respiratory supercomplexes” are situated at the relatively planar membrane regions.

These intriguing questions may now be answered with the author’s transmembrane electrostatic proton localization theory^27–31^. Recently, we introduced a new protonic motive force (pmf) equation^29^ to account for the effect of localized protons^31–39^ at a liquid-membrane interface in mitochondria, bacteria and other biological systems. For example, with a preliminary application of the transmembrane electrostatic proton localization theory^27–31^, we were recently able to show a large enough pmf to synthesize ATP in alkalophilic bacteria^28,40^, elucidating the decades-longstanding bioenergetic conundrum^41–44^ on how the bacteria can synthesize ATP for cell growth^45–47^. We have also experimentally demonstrated the creation of a transmembrane electrostatically localized proton layer at the water-membrane interface in a biomimetic anode water-membrane-water cathode system^34^.

Our work^29,31,39^ has also explained that the Gouy-Chapman theory^48^ and the Debye length concept are not applicable to determining the thickness of localized excess proton layer. The reason is that the Debye length equations are defined for charge-balanced solutions including 1:1 electrolyte solutions such as NaCl solution, but not for the special localized excess protons^49^. Furthermore, the mitochondrial inner membrane is an insulator, which is very different from an electrode that the “electric double layer” and Gouy-Chapman theory^48^ may apply. Consequently, the transmembrane electrostatically localized excess proton layer at the liquid-membrane interface likely is a special monolayer (with a thickness about 1 nm), but definitely not an “electric double layer” that would be predicted by the Gouy-Chapman theory^48^ at a charged electrode-liquid interface. This conclusion as a localized excess proton monolayer at the liquid-membrane interface is also in line with the known “electric double layer” phenomenon in that the excess proton layer may be regarded as an extension from the secondary (proton) layer of the anode’s “electric double layer” around the liquid water body surface as reported in our previous experimental study^39^.

Cardiolipin (1,3-bis(*sn*-3′-phosphatidyl)-*sn*-glycerol) was once believed to play a role in the “localized proton microcircuits”^38,50^. If its phosphate-associated hydrophilic head group indeed serves as a proton “trap” or a protonic conduction pathway at the liquid-membrane interface along the membrane surface, as previously proposed by Haines and Dencher^51^, that would certainly expose its “anhydrous” protons to the bulk aqueous phase. Therefore, it is not realistic for such cardiolipin “localized proton microcircuits” to function. As we can expect from the transmembrane electrostatic proton localization theory^27–31^ that cardiolipin is not required for the formation of protonic capacitor^28,29,39^, cardiolipin is indeed dispensable for oxidative phosphorylation as recently confirmed by an independent experimental study^52^. We have recently also clarified that the protons and ions of any permanent “electric double layer” formed as a result of membrane surface’s fixed-charges such as the negatively-charged phosphate groups of any phospholipids including cardiolipin that permanently attract protons and ions are irrelevant to the membrane potential $\triangle {\psi }$ in proton-coupling bioenergetics^29^. This is also well in line with the conclusion from our previous study^53^: “membrane surface-fixed-charges-attracted protons are not relevant to the protonic motive force” that drives ATP synthesis.

More recently, employing the Lee transmembrane electrostatic proton localization theory^27,28,30,31^ with a set of new equations for pmf that takes electrostatically localized protons into account, we have now achieved much better understanding of membrane potential $\triangle {\psi }$ as the voltage difference contributed by the localized surface charge density at the liquid-membrane interface as in “a transmembrane electrostatically localized protons/cations-membrane-anions capacitor”^29^.

In this article, to answer the fundamental questions on the biological significance of mitochondrial cristae formation, the transmembrane electrostatic proton localization theory^28–31^ is now further employed in calculating the numbers of electrostatically localized protons at the liquid-membrane interface per mitochondrion. Furthermore, the geometric effect of cristae on enhancing localized proton density at the cristae tips is analyzed using an ellipsoidal-shaped mitochondrial crista as a protonic capacitor in accordance with the Lee transmembrane electrostatic proton localization theory^27–31^. This work enables much better understanding of mitochondrial cristae (Fig. 1) in relation to localized proton coupling bioenergetics. The biological significance of mitochondrial cristae formation in regarding to why the dimers of F_1_F_o_-ATP synthase are distributed primarily at the mitochondrial cristae tips whereas the proton-pumping “respiratory supercomplexes” are situated at the planar membrane regions is now elucidated with protonic bioenergetics.

## Transmembrane Electrostatic Proton Localization Theory and Calculations

### The transmembrane electrostatic proton localization theory

**Figure 2:**
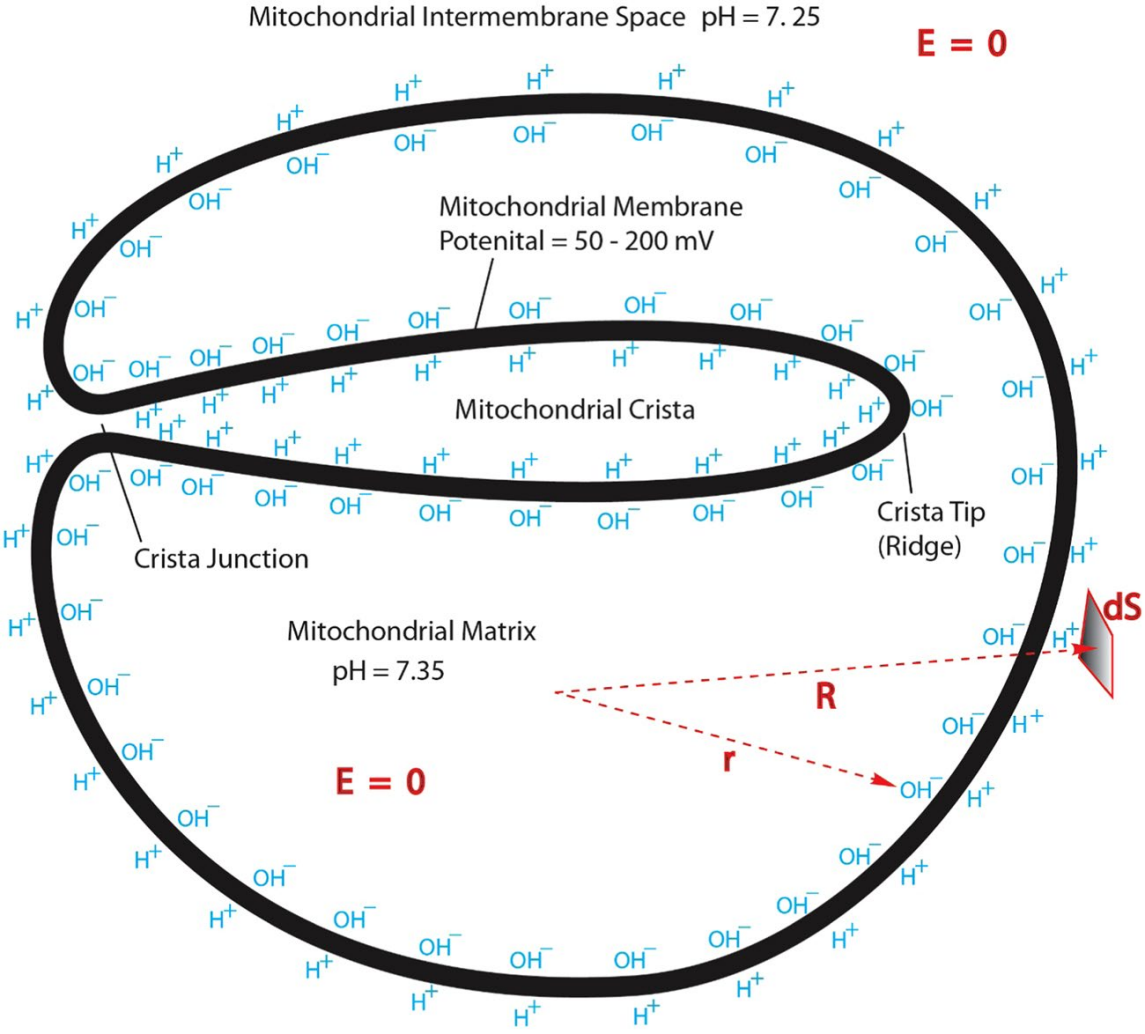
Cross section for a mitochondrion with crista: transmembrane electrostatic proton localization (protonic capacitor) model for a mitochondrion with crista illustrating how excess protons (H^+^) and hydroxyl ions (OH^–^) could be electrostatically localized at the water-membrane interfaces along the two sides of the mitochondrial inner membrane with crista formation before proton-cation exchange as it would be in a theoretically pure water-membrane-water system.

This novel proton-coupling bioenergetics theory^27–31,54^ is built on the fundamental understanding that a biologically relevant water liquid phase can act as a protonic conductor. This new understanding is well in line with the well-established knowledge that protons can quickly transfer among water molecules by the “hops and turns” mechanism^55–57^. That is, liquid water can serve as a protonic conductor because protons using the “hops and turns” mechanism can translocate quickly among water molecules. Notably, from the negative charge (“proton hole”) perspective, hydroxyl anions (“proton holes”) translocate among the water molecules in the opposite direction of protonic conduction. Consequently, excess protons (positive charges) in an aqueous liquid on one side of a membrane will repel each other to become electrostatically localized along the membrane surface, attracting an equal number of excess hydroxyl anions (negative charges) to the other side of the membrane and thus resulting in a “protonic capacitor structure” (Fig. 2).

As previously reported^27–29^, this protonic capacitor may be mathematically justified employing the Gauss’ Law and the knowledge that the electric field **E** is zero inside a protonic conductor. Although the Gauss’ Law has recently been applied to bacteria^28^ and a simplified mitochondrion^29^ without cristae, it has never been done to a mitochondrion with a crista or cristae before. Therefore, Fig. 2 represents the application of the Gauss’ Law to a mitochondrion with a crista for the first time. This application and its result are not entirely obvious. However, it can be justified according to the knowledge that the mitochondrial crista liquid is connected through the crista junction with the liquid in the mitochondrial intermembrane space (Fig. 2).

As described in refs. ^58^ and^29^, Gauss’ Law is known to relate the net charge Q within a volume to the flux of electric field lines through the closed surface surrounding the volume by the following integral equation

1 $${{\rm{\varepsilon }}}_{{\rm{o}}}\,\oint {\bf{E}}\cdot {\bf{d}}{\bf{S}}={\rm{Q}}$$

where ε_o_ is the electric permittivity constant; **E** is the electric field inside a protonic conductor; and **dS** is a differential surface element. In this equation (Eq. 1), the small circle on the integral sign indicates that the integration is performed over the closed surface.

So, let’s now consider a series of integration applications using the Gauss’ Law equation (Eq. 1), where a small volume near the center of the mitochondrial matrix liquid is gradually increased until it is just inside the matrix liquid surface as indicated by a polar coordinate **r** in Fig. 2. Since the electric field **E** is zero everywhere in a conductive liquid body, the left side of Eq. 1 vanishes in each case. Consequently, the right side of the equation (Eq. 1) must also vanish, which means that no net charge (Q = 0) is within the volume of the mitochondrial matrix liquid phase; the excess hydroxyl anions in this case must therefore be at the matrix liquid-body surface, i.e. at the liquid-membrane interface along the matrix (*n*) side of mitochondrial inner membrane surface.

Similarly, considering the proton-conductive aqueous liquid phase at the mitochondrial intermembrane space including cristae space, the electric field **E** = 0 is true at anywhere within the bulk aqueous liquid phase of both the intermembrane space and the crista space that are connected through a crista junction (Fig. 2). Employing the Gauss’ Law integral equation to a series of volumes enclosing the entire mitochondrion and reducing them to be just outside its inner membrane surface (including its cristae membrane surface) as illustrated by a polar coordinate **R** in Fig. 2, the surface integrals of Eq. 1 in this case also equal to zero so that no net excess charge is found. Since the previous application of Gauss’ Law equation (Eq. 1) in the matrix liquid already informed us that the excess hydroxyl anions are on the inner membrane surface at the matrix (*n*) side, the positive charges (excess protons) must be at the membrane-liquid interface along the membrane surface at the intermembrane/cristae space (*p*) side (including cristae junctions and cristae liquid space side). As a result, the localized positive charges (excess protons) at the membrane-liquid interface on the intermembrane/cristae space (*p*) side precisely balances the excess hydroxyl anions on the matrix (*n*) side, making the total net charge of the entire mitochondrion zero. This mathematically justifies that a mitochondrion with cristae is also a protonic membrane capacitor.

### New protonic motive force equation with protonic capacitor

The transmembrane electrostatic proton localization theory^27–31,53^ and the successful experimental demonstration of a protonic capacitor using biomimetic “water-membrane-water” systems^34,39,54,59^ clearly indicate that the textbook Mitchellian chemiosmotic theory is not entirely correct because the Mitchellian delocalized proton coupling view treats protons pretty much like typical solutes in bulk liquid phases and thus fails to consider the existence of localized proton coupling. Consequently, the textbook Mitchellian pmf equation quite fatally misses a major pmf contribution from the electrostatically localized protons at the liquid-membrane interface that is rightly at the ATP synthase protonic inlet mouth. Therefore, the textbook Mitchellian pmf equation must now be revised. It is now quite clear that the population density of localized excess protons is immediately related to the probability for protons to be available at the protonic mouth of ATP synthase, in addition to that implicated from the bulk liquid phase pH. Therefore, based on the new bioenergetics understanding, the author^29,40^ has recently rewritten the protonic motive force (pmf) equation for ATP synthesis as

2 $${\rm{pmf}}\,=\Delta \psi +\frac{2.3\,RT}{F}{\log }_{10}([{H}_{pB}^{+}]/[{H}_{nB}^{+}])+\frac{2.3\,RT}{F}{\log }_{10}(1+[{H}_{L}^{+}]/[{H}_{pB}^{+}])$$

where $\triangle {\psi }$ represents the transmembrane electrical potential difference (positive (*p)*-phase minus negative (*n)*-phase defined as in ref. ^60^); *R* is the gas constant; *T* is the absolute temperature; *F* is Faraday’s constant; $[{H}_{pB}^{+}]$ is the *p*-side bulk liquid phase proton concentration in the intermembrane space including cristae space; $[{H}_{nB}^{+}]$ is the proton concentration in mitochondrial matrix (*n*) bulk liquid phase; and $[{H}_{L}^{+}]$ is the effective concentration of electrostatically localized protons at the liquid-membrane interface along the positive (*p*) side of the mitochondrial inner membrane.

As pointed out previously^29,40^, the first two terms of Eq. 2 represent the Mitchellian bulk phase-to-bulk phase proton electrochemical potential gradient for which we now call it as the “classic” pmf; whereas the last term represents the “local” pmf from the electrostatically localized protons.

For an idealized protonic capacitor, as described previously^29,40^, the ideal localized proton concentration ${[{H}_{L}^{+}]}^{0}$ at the water–membrane interface on the *p*-side can be expressed as a function of the transmembrane electrical potential difference Δ*ψ* in the following equation

3 $${[{H}_{L}^{+}]}^{0}=\frac{C}{S}\cdot \frac{\Delta \psi }{l\cdot F}$$

where $C/S$ represents the specific membrane capacitance per unit surface area; and $l$ is the thickness of the localized proton layer.

In real biological systems such as mitochondria, the non-proton cations ${M}_{pB}^{i+}$ (for examples, Na^+^, K^+^, and Mg^2+^) of the bulk liquid *p*-phase in the intermembrane space including cristae space may exchange with the localized protons at the liquid-membrane interface and thereby reduce the localized proton concentration. As explained in our recent study^29^, for any given number (n) of different non-proton cation species such as Na^+^, K^+^, and Mg^2+^ present in the bulk aqueous *p*-phase of the intermembrane space including cristae space, the localized proton concentration $\,[{H}_{L}^{+}]$ after exchanging with all of the cation species at the equilibrium state can be expressed as

4 $$[{H}_{L}^{+}]=\frac{{[{H}_{L}^{+}]}^{0}}{{\prod }_{i=1}^{n}\,\{{K}_{Pi}\left(\frac{[{M}_{pB}^{i+}]}{[{H}_{pB}^{+}]}\right)+1\}}$$

Here $[{M}_{pB}^{i+}]$ is the concentration of any given non-proton cation in the bulk liquid *p*-phase of the intermembrane space including cristae space; and $\,{K}_{Pi}$ is the equilibrium constant for each of the cation species to exchange with the localized protons. As pointed out previously^29,40^, the denominator in Eq. 4 is the reduction factor owing to the effect of cation exchange with the localized protons at the liquid-membrane interface along the inner membrane surface at the intermembrane/cristae space (*p*) side (Fig. 2). The total contribution of the non-proton cations to this reduction factor surprisingly is a product (multiplication not summation) of the reduction factors associated with each of the non-proton cation species $[{M}_{pB}^{i+}]$ in the bulk liquid *p*-phase such as Na^+^, K^+^, and Mg^2+^ at the intermembrane space including the cristae space.

### Newly formulated membrane potential equations with protonic capacitor

Another deficiency of the classic Mitchellian chemiosmotic theory is that it does not clearly explain the biophysical origin of membrane potential ∆ψ. Mitchell and his coworker (1969) once stated “ Δ*ψ* can be equated to the Donnan potential”^61^ which seems vaguely regarding membrane potential Δ*ψ* as “a delocalized parameter” somehow contributed by delocalized protons and ions in the two bulk liquid phases^60^. As shown in our recent publication^29^ and illustrated here with the mitochondrial proton-electrostatics localization model (Fig. 2), the localized excess protons and hydroxyl ions along the two sides of the membrane may directly contribute to the trans-membrane potential difference ∆ψ; In contrast, the Mitchellian “delocalized protons” that can be measured by the bulk liquid phase pH have little to do with the membrane potential Δ*ψ*. Consequently, the membrane potential (difference) Δ*ψ* created by the localized proton concentration ${[{H}_{L}^{+}]}^{0}$ before cation exchange with the localized protons is now expressed as in the following protonic capacitor-based Δ*ψ* equation reported previously^29^:

5 $$\Delta \psi =\frac{S\cdot l\cdot F\cdot {[{H}_{L}^{+}]}^{0}}{C}$$

where $C/S$ is the specific membrane capacitance per unit surface area, $l$ is the thickness of the localized proton layer, and $F$ is the Faraday constant.

From the protonic capacitor-based membrane potential Δ*ψ* equation (Eq. 5) as reported in our recent study^29^, we can now clearly understand that the membrane potential (difference) Δ*ψ* is a function of the ideal localized proton concentration ${[{H}_{L}^{+}]}^{0}$. This also elucidates the physical origin of membrane potential Δ*ψ* as a function of the localized excess proton concentration at the liquid-membrane interface under the idealized condition.

In actual biological systems such as animal mitochondria, as discussed previously^29^, the cation-proton exchange will reduce the localized proton concentration from ${[{H}_{L}^{+}]}^{0}$ to $\,[{H}_{L}^{+}]$; but it typically does not change the membrane potential. Therefore, the relationship between ${[{H}_{L}^{+}]}^{0}$ and $[{H}_{L}^{+}]$ may be expressed with the product of cation exchange reduction factors as

6 $${[{H}_{L}^{+}]}^{0}=[{H}_{L}^{+}]\cdot \mathop{\prod }\limits_{i=1}^{n}\{{K}_{Pi}\left(\frac{[{M}_{pB}^{i+}]}{[{H}_{pB}^{+}]}\right)+1\}$$

By combining Eqs. 5 and 6, the membrane potential difference Δ*ψ* at the cation-proton exchange equilibrium may be expressed as

7 $$\Delta \psi =\frac{S\cdot l\cdot F\cdot [{H}_{L}^{+}]\cdot {\prod }_{i=1}^{n}\{{K}_{Pi}\left(\frac{[{M}_{pB}^{i+}]}{[{H}_{pB}^{+}]}\right)+1\}}{C}$$

As reported previously^29^, since the cation-proton exchange typically does not change the total localized (positive) charge density at the liquid-membrane interface, the ideal localized protons ${[{H}_{L}^{+}]}^{0}$ equals to the sum of the localized proton concentration $[{H}_{L}^{+}]\,$ and the localized cation concentrations ($\mathop{\sum }\limits_{i=1}^{n}[{M}_{L}^{i+}]$) at the cation-proton exchange equilibrium as shown in the following equation.

8 $${[{H}_{L}^{+}]}^{0}=[{H}_{L}^{+}]+\mathop{\sum }\limits_{i=1}^{n}[{M}_{L}^{i+}]$$

Where $[{M}_{L}^{i+}]$ is the concentration for each of the localized non-proton cations such as sodium and potassium cations in cation-proton exchange equilibrium at a liquid-membrane interface. Therefore, as concluded previously^29^, the membrane potential difference Δ*ψ* can also be expressed as

9 $$\Delta \psi =\frac{S\cdot l\cdot F\cdot ([{H}_{L}^{+}]+{\sum }_{i=1}^{n}[{M}_{L}^{i+}])}{C}$$

This protonic/cationic capacitor-based Δ*ψ* equation (Eq. 9) also elucidates the physical origin of membrane potential (difference) Δ*ψ* as a function of the localized proton concentration $[{H}_{L}^{+}]$ and localized cation concentrations $\mathop{\sum }\limits_{i=1}^{n}[{M}_{L}^{i+}]$ in mitochondrial membrane system at cation-proton exchange equilibrium. That is, the membrane potential Δ*ψ* is dependent on the transmembrane electrostatically localized protons $[{H}_{L}^{+}]$ and cations $\mathop{\sum }\limits_{i=1}^{n}[{M}_{L}^{i+}]$ at the liquid-membrane interface along the *p*-side surface of the mitochondrial inner membrane as expressed in Eq. 9. Therefore, the membrane potential Δ*ψ* in mitochondria can now be understood as a localized protonic/cationic membrane capacitor behavior^29^.

### Calculations on total cation exchange reduction factor and electrostatically localized proton concentration

As reported previously^29^, the total cation exchange reduction factor as shown in the denominator of Eq. 4 was calculated from the concentrations of the major cations $[{M}_{pB}^{i+}]$, their cation-proton exchange equilibrium constants *K*_*Pi*_ and the *p*-side bulk liquid phase proton concentration $[{H}_{pB}^{+}]$ in the mitochondrial intermembrane space and cristae space. Briefly, the concentrations of the major cations 10 mM Na^+^, 128 mM K^+^, and 1.0 mM Mg^2+^ in the mitochondria were calculated from the composition of the reaction medium reported in ref. ^62^. The cation-proton exchange equilibrium constants $\,{K}_{Pi}$ for Na^+^ and K^+^ were 5.07 ×10^−8^ and 6.93 ×10^−8^, respectively, as determined previously by experimental measurements^39^. The $\,{K}_{Pi}$ value of 5.42 ×10^−6^ for Mg^2+^ was determined from the latest experimental data of divalent cation Mg^++^ exchange with electrostatically localized protons at a membrane-liquid interface in the Lee laboratory through a study similar to that reported in ref. ^39^.

Chinopoulos *et al*.^62^ elegantly revealed that the bulk-phase pH difference across the mitochondrial inner membrane actually is near zero: the “∆pH_max_ is only ~0.11”. This observation, as discussed previously^29^, is well corroborated with our recent biomimetic experimental demonstration that, before and after the membrane is energized with excess protons at the anode side of the membrane and excess hydroxyl anions at the cathode side of the membrane, the bulk-phase liquid pH in the anode liquid chamber remained about the same as that in the cathode chamber liquid^34,39,54,59^. By this corroboration, therefore, it is well justified to use the experimental data from Chinopoulos *et al.*^62^ in regarding to the mitochondria reaction medium pH 7.25 (*pH*_*pB*_) and matrix liquid pH 7.35 (*pH*_*nB*_) for our study here as well. Accordingly, the total cation exchange reduction factor (the denominator of Eq. 4) was calculated to be 1.29, indicating a relatively minor role in modulating the electrostatically localized surface proton concentration by the cation-proton exchange process in mitochondria. That is, the electrostatically localized proton concentration $[{H}_{L}^{+}]$ at the cation-proton exchange equilibrium state represent about 78% of the total localized surface charge density in animal mitochondria.

For a mitochondrial membrane potential Δ*ψ* in a range from 50 to 200 mV, as reported previously^29^, the ideal localized proton population density ${[{H}_{L}^{+}]}^{0}$ (before the cation-proton exchange process) was calculated to be in a range from 6.84 to 27.4 mM, which is now known to equal with the total localized surface charge density: the sum of the localized protons $[{H}_{L}^{+}]$ and the localized non-proton cations $\mathop{\sum }\limits_{i=1}^{n}[{M}_{L}^{i+}]$. Depending on the membrane potential Δ*ψ*, the concentration of electrostatically localized protons $[{H}_{L}^{+}]$ at the liquid-membrane interface in animal mitochondria was calculated to be in a range from about 5.30 to 21.2 mM at the cation-proton exchange equilibrium. Using the data of electrostatically localized protons $[{H}_{L}^{+}]$ and mitochondrial inner membrane surface area ($S$), the amounts of localized protons per mitochondrion can now be calculated for mitochondria with and without cristae using Eqs. 10 and 11 described as follows.

## Results and Discussions

### Formation of cristae creating more mitochondrial inner membrane surface area and protonic capacitance

From the protonic membrane capacitor of Fig. 2, it is quite apparent that mitochondrial cristae formation creates more mitochondrial inner membrane surface area ($S$), which immediately contributes to its membrane capacitance ($C$). Therefore, its bioenergetics significance can now be better understood with the Lee model^28–31,54^ of transmembrane electrostatically localized protons as shown in the membrane potential (Δ*ψ*) equation (Eq. 5), which shows that Δ*ψ* exists precisely because of the excess cations (including H^+^) and the excess anions (such as OH^−^) charge layers localized on the two sides of the membrane in a protons-membrane-anions capacitor structure (Fig. 2). This also explains the origin of Δ*ψ* and its relationship with the concentration of electrostatically localized protons $[{H}_{L}^{+}]$ as expressed in Eqs. 3 and 4.

**Figure 3:**
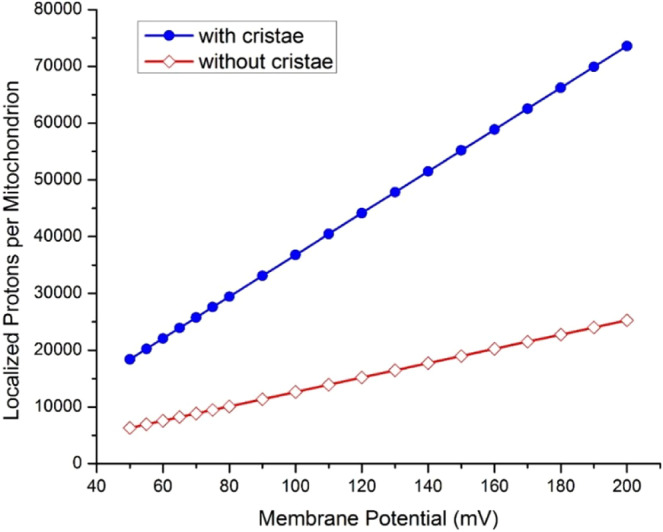
Comparing the numbers of transmembrane electrostatically localized protons per mitochondrion for a mitochondrion (in a size of 1500 nm × 300 nm × 300 nm) without cristae (open diamond red) and with cristae (filled circle blue) as a function of membrane potential (mV).

**Figure 4:**
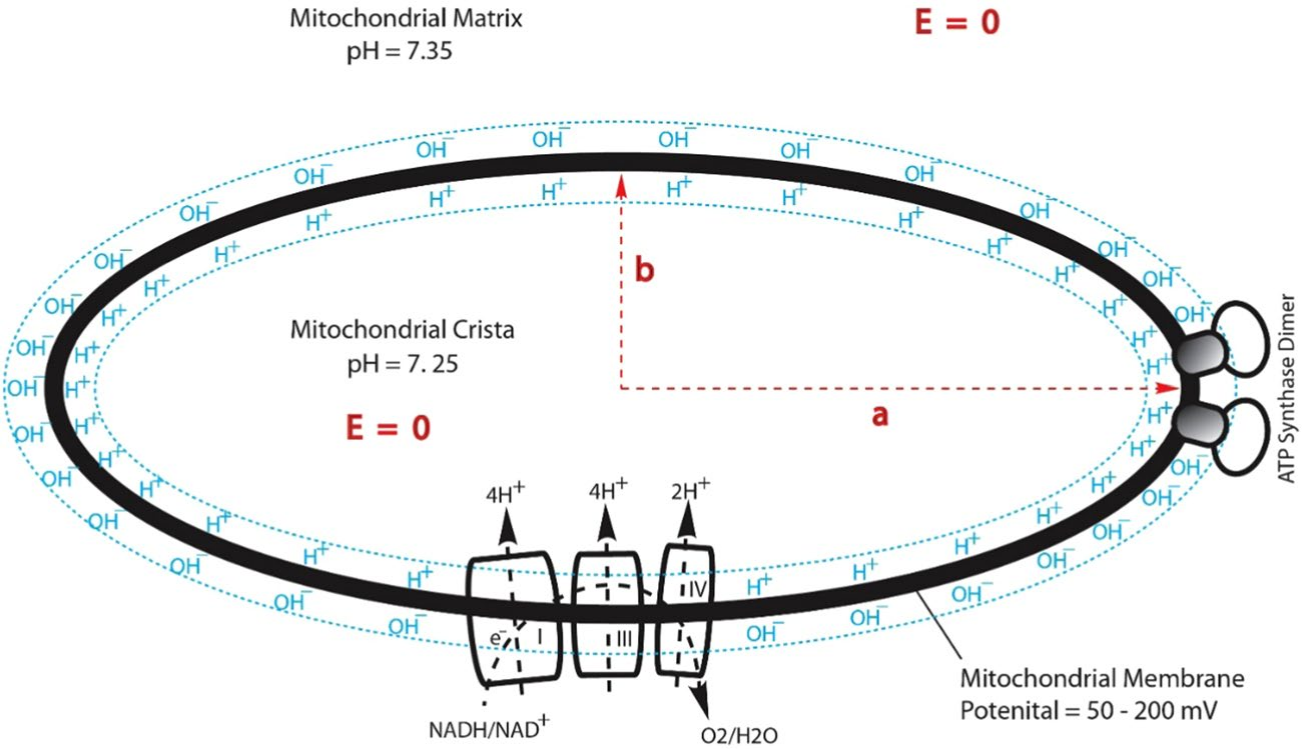
A cross section for an ellipsoidal-shaped mitochondrial crista: transmembrane electrostatic proton localization (protonic capacitor) model illustrating how excess protons (H^+^) and hydroxyl ions (OH^–^) could be electrostatically localized at the water-membrane interfaces along the two sides of the mitochondrial inner membrane before proton-cation exchange as it would be in a theoretically pure water-membrane-water system. Note, this cross section can be considered as a special result from the tri-axial (a, b, and c) protonic conducting ellipsoidal crista equation (Eq. 12) in 3-dimensional x, y, and z coordinates for its middle cross section (where the z coordinate is zero).

From the membrane potential Δ*ψ* equation (Eq. 9), it is now also quite clear that the amount of transmembrane electrostatically localized charges (*C* · Δ*ψ*) per mitochondrion may be calculated from mitochondrial inner membrane surface area (*S*) and localized proton and cation concentrations $\,([{H}_{L}^{+}]+\mathop{\sum }\limits_{i=1}^{n}[{M}_{L}^{i+}])$ as

10 $$Localized\,charges=C\cdot \Delta \psi =S\cdot l\cdot F\cdot ([{H}_{L}^{+}]+\mathop{\sum }\limits_{i=1}^{n}[{M}_{L}^{i+}])$$

From here, we can also understand, the amount of transmembrane electrostatically localized charges per mitochondrion is contributed by both the mitochondrial inner membrane capacitance ($C$) and membrane potential (Δ*ψ*). The mitochondrial inner membrane capacitance ($C$) is intimately related to the membrane area ($S$). Therefore, when a mitochondrion creates more inner membrane surface area ($S$) through cristae formation, its capacitance ($C$) increases and thus enhances its ability to store transmembrane electrostatically localized charges (energy). This also explains part of the biological significance in mitochondrial cristae formation.

Similarly, when the data of inner membrane surface area ($S$), the localized proton layer thickness ($l$), and localized proton concentration $[{H}_{L}^{+}]$ are available, the amounts of transmembrane electrostatically localized protons per mitochondrion may be calculated for mitochondria with and without cristae according to the following equation:

11 $$Localized\,protons=S\cdot l\cdot {N}_{A}\cdot [{H}_{L}^{+}]$$

where ${N}_{A}$ is the Avogadro constant (6.02205 × 10^23^ mol^−1^).

### Transmembrane electrostatically localized protons at the liquid-membrane interface per mitochondrion calculated as a function of membrane potential

Table 1 lists the numbers of transmembrane electrostatically localized protons per mitochondrion (in a size about 1500 nm × 300 nm × 300 nm) with and without cristae calculated as a function of transmembrane potential Δ*ψ* using Eqs. 3, 4 and 11 under the given reaction medium pH 7.25 ($p{H}_{pB}$), mitochondria matrix pH 7.35 ($p{H}_{nB}$) and taking cation-proton exchange into account.

**Table 1:** Transmembrane electrostatically localized protons per mitochondrion (in a size about 1500 nm × 300 nm × 300 nm) with and without cristae calculated as a function of transmembrane potential Δ*ψ* using Eqs. 3, 4 and 11 under the given reaction medium pH 7.25 ($p{H}_{pB}$), mitochondria matrix pH 7.35 ($p{H}_{nB}$), specific membrane capacitance ($C/S$) of 13.2 mf/m^2^ based on measured experimental data^64^, localized proton layer thickness ($l$) of 1 nm as discussed in ref. ^28,34^, exchange reduction factor of 1.29 as calculated previously^29^, and inner membrane surface area of 5.76 ×10^6^ nm^2^ for the mitochondrion with cristae and 1.98 ×10^6^ nm^2^ for the mitochondrion without any cristae.

Δ*ψ* (mV)	Localized protons per mitochondrion with cristae	Localized protons per mitochondrion without cristae
50	1.84 × 10^4^	6.32 × 10^3^
55	2.02 × 10^4^	6.96 × 10^3^
60	2.21 × 10^4^	7.59 × 10^3^
65	2.39 × 10^4^	8.22 × 10^3^
70	2.58 × 10^4^	8.85 × 10^3^
75	2.76 × 10^4^	9.48 × 10^3^
80	2.94 × 10^4^	1.01 × 10^4^
90	3.31 × 10^4^	1.14 × 10^4^
100	3.68 × 10^4^	1.26 × 10^4^
110	4.05 × 10^4^	1.39 × 10^4^
120	4.41 × 10^4^	1.52 × 10^4^
130	4.78 × 10^4^	1.64 × 10^4^
140	5.15 × 10^4^	1.77 × 10^4^
150	5.52 × 10^4^	1.90 × 10^4^
160	5.89 × 10^4^	2.02 × 10^4^
170	6.25 × 10^4^	2.15 × 10^4^
180	6.62 × 10^4^	2.28 × 10^4^
190	6.99 × 10^4^	2.40 × 10^4^
200	7.36 × 10^4^	2.53 × 10^4^

According to a 3-dimensional mitochondrial cristae model of an intermyofibrillar mitochondrion with a size about 1500 nm × 300 nm × 300 nm^63^, the surface area of mitochondrial inner membrane is 5.76 × 10^6^ nm^2^, which is about three times larger than the surface area of the mitochondrial outer membrane (1.98 × 10^6^ nm^2^). Using the inner membrane surface area of 5.76 × 10^6^ nm^2^ for a typical mitochondrion with cristae, the numbers of electrostatically localized protons per mitochondrion were calculated through Eq. 11 to be in a range from 1.84 × 10^4^ to 7.36 × 10^4^ depending on the membrane potential Δ*ψ* in a range from 50 to 200 mV (Table 1 and Fig. 3). On the other hand, assuming 1.98 × 10^6^ nm^2^ as the inner membrane surface area for a mitochondrion without any cristae, the numbers of electrostatically localized protons per mitochondrion were calculated to be in a range from 6.32 × 10^3^ to 2.53 × 10^4^ depending on the membrane potential Δ*ψ* (Table 1 and Fig. 3).

By comparing the numbers of electrostatically localized protons for mitochondria with and without cristae as presented in Fig. 3, it is now quite clear that a mitochondrion with cristae has as many as nearly 3-times more electrostatically localized protons which can be used to drive ATP synthesis than a mitochondrion without any cristae. Therefore, we can now understand that mitochondrial cristae formation may favor the energy transduction process through transmembrane electrostatically localized protons for ATP synthesis. That is, the present study with this finding (Table 1 and Fig. 3) may represent a significant step forward with the protonic bioenergetics as a complementary development to the early empirical understanding such as “the surface area of cristae positively correlates with the amount of ATP produced by oxidative phosphorylation in various tissues” as stated by Zick *et al*. in a review article^1^.

Consequently, the data in Fig. 3 indicate that, at least, one of the biological significances in the formation of cristae is to create more mitochondrial inner membrane surface area and thus more protonic capacitance for localized proton energy storage. This may represent a type of quantitative (optional) improvement that is biologically advantageous, but not necessarily an absolute requirement for all other organisms. It may also explain why seem only mitochondria have cristae formed so extensively; while some bacteria have an intracytoplasmic membrane structure similar to cristae and others may or may not have such a structure^65^.

### Geometric effect of a crista enhancing transmembrane-electrostatically localized proton density at the crista tip

As shown in Fig. 1, a mitochondrial crista may be considered as an ellipsoidal-shaped 3D-structure^66–69^ which can be described by a tri-axial (a, b, and c) ellipsoid equation (Eq. 12) in 3-dimensional x, y, and z coordinates. As illustrated in Fig. 4 for a middle cross section of an ellipsoidal-shaped mitochondrial crista, the complexes I, III and IV that constitute the “respiratory supercomplexes” are situated at the relatively flat membrane regions, utilizing NADH as the source of electrons and molecular oxygen (O2) as the terminal electron acceptor and pumping protons across the inner membrane from the mitochondrial matrix into the crista liquid space. The excess protons in the crista liquid space created by the redox-driven proton pumps distribute themselves electrostatically to the liquid-membrane interface at the crista liquid side of the inner membrane. The ATP synthase as its dimer located at the crista tip (ridge) utilizes the transmembrane-electrostatically localized protons there to drive ATP synthesis from ADP and Pi. We now understand, the feature that ATP synthase as its dimer located at the crista tip (ridge) has an advantage in relation to protonic bioenergetics, since the localized proton concentration there is higher than that at the relatively flat membrane regions as shown in the following analysis.

As shown in Fig. 4, the electric field **E** = 0 in both mitochondrial matrix and crista indicates that both the matrix and crista liquids can function as protonic conductors in accordance with the Lee transmembrane-electrostatic electrostatic proton localization theory^27–31^. In recognizing the mitochondrial crista liquid as protonic conductor, based on the mathematic solution of conducting ellipsoid and circular disk electrostatics developed by Prof. Kirk McDonald of Princeton University^70^, we can now understand that the surface protonic charge density σ on a tri-axial (a, b, and c) protonic conducting ellipsoidal crista in 3-dimensional x, y, and z coordinates

12 $$\frac{{x}^{2}}{{a}^{2}}+\frac{{y}^{2}}{{b}^{2}}+\frac{{z}^{2}}{{c}^{2}}=1$$

can be written as

13 $${{\rm{\sigma }}}_{ellipsoidalcrista}=\frac{Q}{4\pi abc\sqrt{\frac{{x}^{2}}{{a}^{4}}+\frac{{y}^{2}}{{b}^{4}}+\frac{{z}^{2}}{{c}^{4}}}}$$

where $Q$ is the total charge of electrostatically localized protons; and $a$, $b$ and $\,c$ are the ellipsoid axial lengths with respect to the 3-dimensional x, y, and z coordinates.

At the crista tip where the ATP synthase dimer is located, the coordinates are: x = a, y = 0, and z = 0 (a, 0, 0). Therefore, the localized protonic charge density at the crista tip is

14 $${{\rm{\sigma }}}_{tip}=\frac{Q}{4\pi abc\sqrt{\frac{{a}^{2}}{{a}^{4}}\,+\frac{{0}^{2}}{{b}^{4}}+\frac{{0}^{2}}{{c}^{4}}}}=\frac{Q}{4\pi bc}$$

Similarly, at the crista flat region where the coordinates are: x = 0, y = b, and z = 0 (0, b, 0), the localized protonic charge density is

15 $${{\rm{\sigma }}}_{flat}=\frac{Q}{4\pi abc\sqrt{\frac{{0}^{2}}{{a}^{4}}\,+\frac{{b}^{2}}{{b}^{4}}+\frac{{0}^{2}}{{c}^{4}}}}=\frac{Q}{4\pi ac}$$

Consequently, the ratio of the localized protonic charge density at the crista tip to that at the crista flat region is

16 $$\frac{{{\rm{\sigma }}}_{tip}}{{{\rm{\sigma }}}_{flat}}=\frac{\frac{Q}{4\pi bc}}{\frac{Q}{4\pi ac}}=\frac{\,a\,}{b}$$

Note, the crista localized protonic charge density (${{\rm{\sigma }}}_{crista}$) with the thickness ($l$) of localized proton layer is related to the crista localized proton concentration (${[{H}_{L}^{+}]}_{crista}^{0}$):

17 $${[{H}_{L}^{+}]}_{crista}^{0}=l\cdot {{\rm{\sigma }}}_{crista}$$

Therefore, the ratio of the localized proton concentration at the crista tip (${[{H}_{L}^{+}]}_{tip}^{0}$) to that at the crista flat region (${[{H}_{L}^{+}]}_{flat}^{0}$) is the same as the ratio of the localized protonic charge density at the crista tip to the flat region, which equals to the axial ratio ($a/b$):

18 $$\frac{{[{H}_{L}^{+}]}_{tip}^{0}}{{[{H}_{L}^{+}]}_{flat}^{0}}=\frac{{{\rm{\sigma }}}_{tip}}{{{\rm{\sigma }}}_{flat}}=\frac{\,a\,}{b}$$

This is an important finding, since it mathematically shows that the formation of a crista may enhance the localized proton concentration at the crista tip (ridge) relatively to the flat region of a crista because of the geometric effect of protonic electrostatics.

Table 2 lists the calculated ratios of the transmembrane electrostatically localized proton concentration at the crista tip (${[{H}_{L}^{+}]}_{tip}^{0}$) to that at the crista flat region (${[{H}_{L}^{+}]}_{flat}^{0}$) in relation to the axial ratios ($a/b$) associated with various ellipsoidal cristae lengths (2 $a$) and widths (2 $b$). The results showed that it is the ratio of ellipsoidal cristae length (2 $a$) to its width (2 $b$) that determines the geometric effect of cristae on electrostatically localized proton density. For example, the ratios of the transmembrane electrostatically localized proton concentration at the crista tip (${[{H}_{L}^{+}]}_{tip}^{0}$) to that at the crista flat region (${[{H}_{L}^{+}]}_{flat}^{0}$) can be anywhere in a range from 1 up to 15 depending on the axial ratio ($a/b$) of the ellipsoidal cristae length to its width. That is, the localized proton concentration at the crista tip (${[{H}_{L}^{+}]}_{tip}^{0}$) can be significantly higher than that at the crista flat region (${[{H}_{L}^{+}]}_{flat}^{0}$) depending on the geometric effect of cristae in protonic electrostatics.

**Table 2:** Geometric effect of cristae on transmembrane electrostatically localized proton density at the cristae tips (ridges) in relation to the axial ratios ($a/b$) associated with the ellipsoidal cristae length and width.

Crista length (2 *a*) nm	Crista width (2 *b*) nm	Axial ratio (*a/b*)	$\frac{{[{{\bf{H}}}_{{\bf{L}}}^{{\boldsymbol{+}}}]}_{{\bf{t}}{\bf{i}}{\bf{p}}}^{{\bf{0}}}}{{[{{\bf{H}}}_{{\bf{L}}}^{{\boldsymbol{+}}}]}_{{\bf{f}}{\bf{l}}{\bf{a}}{\bf{t}}}^{{\bf{0}}}}$	$-{\bf{L}}{\bf{o}}{{\bf{g}}}_{{\bf{10}}}\left(\frac{{[{{\bf{H}}}_{{\bf{L}}}^{{\boldsymbol{+}}}]}_{{\bf{t}}{\bf{i}}{\bf{p}}}^{{\bf{0}}}}{{[{{\bf{H}}}_{{\bf{L}}}^{{\boldsymbol{+}}}]}_{{\bf{f}}{\bf{l}}{\bf{a}}{\bf{t}}}^{{\bf{0}}}}\right)$
100	100	1:1	1	0
100	50	2:1	2	−0.30
100	35	2.9:1	2.9	−0.46
100	25	4:1	4	−0.60
100	20	5:1	5	0.70
120	20	6:1	6	−0.78
150	20	7.5:1	7.5	−0.88
200	20	10:1	10	−1.00
240	20	12:1	12	−1.08
260	20	13:1	13	−1.11
280	20	14:1	14	−1.15
300	20	15:1	15	−1.18

As shown in Table, 2, when the axial ratio ($a/b$) is one for a completely round (spherical) membrane system (say, with a diameter of 100 nm), the transmembrane electrostatically localized proton concentration ${[{H}_{L}^{+}]}^{0}$ at the liquid-membrane interface is uniform along the entire spherical membrane surface. On the other hand, when the membrane vesicle system elongates into an ellipsoidal shape such as an ellipsoidal crista with a length of 200 nm and width of 20 nm, the localized proton concentration at the crista tip (${[{H}_{L}^{+}]}_{tip}^{0}$) can be as high as 10 times that at the flat region (${[{H}_{L}^{+}]}_{flat}^{0}$). This translates to a localized proton associated liquid-membrane interface pH difference of about one pH unit between the crista tip (ridge) and the flat region within the same crista. That is, the localized proton associated pH ($-Lo{g}_{10}({[{H}_{L}^{+}]}_{tip}^{0})$) at a crista tip (or ridge) can be significantly lower (by as much as “─ 1.00” pH unit) than that ($-Lo{g}_{10}({[{H}_{L}^{+}]}_{flat}^{0})$) at the flat region within the same crista.

From this result (Table 2), we can now understand that the formation of cristae with ATP synthase dimer rows located at the cristae ridges (tips) where the transmembrane electrostatically localized proton concentration (${[{H}_{L}^{+}]}_{tip}^{0}$) is significantly higher and with the proton-pumping “respiratory supercomplexes” (complexes I, III and V) situated at the relatively flat membrane regions where the localized proton concentration (${[{H}_{L}^{+}]}_{flat}^{0}$) is relatively lower as shown in Fig. 4, is bioenergetically advantageous. That is, at the cristae ridges, the ATP synthases represent a sink (users) for protons, while the proton pumps of the electron transport chain are in the relatively flat membrane plane regions of cristae. Electrostatically guiding the localized protons from their source to the protonic sink at the ATP synthase, the cristae may work as protonic conduits with the liquid-membrane interface along the membrane surface as a transmembrane electrostatically localized protonic conduction pathway (Fig. 4) that enable efficient ATP production.

This finding (Fig. 4, Eq. 18, and Table 2) is remarkably in line with an independent study^11,71^ that “indicate a 3.5-fold increase in surface charge density at the apex, compared with the flat sides” and “the higher surface density of protons in the curved membrane regions would result in a local pH difference of ~0.5 units” for a mitochondrial crista with an axial ratio of 10:1. However, as shown in Table 2, our calculation results from Eq. 18 showed that the ratio of localized proton concentration at the crista tip (${[{H}_{L}^{+}]}_{tip}^{0}$) to that at the flat region (${[{H}_{L}^{+}]}_{flat}^{0}$) is 10:1, translating to a local pH difference of 1 unit, which is twice as high as the “~0.5 units” reported previously^71^ for a mitochondrial crista with an axial ratio of 10:1. Furthermore, based on our protonic capacitor model (illustrated in Figs. 2 and 4, and expressed in Eqs. 3–9), the excess protons are dynamically held at the liquid-membrane interface by the transmembrane-electrostatic proton localization effect^28,29^ without requiring any of the putative interfacial barrier model^72,73^ that the “local proton gradient in the cristae space” model proposed by Davies *et al*.^11^ and presented in a subsequent review article^10^ seems have to invoke; Consistently, our experimentally demonstrated transmembrane-electrostatic excess proton localization at a liquid-membrane interface^39^ also showed “no support” for the putative interfacial barrier model^72,73^ that anyhow is not really required to explain the localized proton coupling bioenergetics. These results all clearly support the suggestion that a position of the ATP synthase at the apex of mitochondrial cristae (as observed in Fig. 1C,D) is optimal for ATP production. This finding (Fig. 4, Eq. 18, and Table 2) is also well corroborated by another independent experimental observation^33^ with bioengineered fluorescent protein as pH indicator that elegantly showed “lateral pH gradient between OXPHOS complex IV and F_o_F_1_ ATP-synthase in folded mitochondrial membranes”.

Therefore, the data in Table 2 indicate another biological significance for cristae formation: the geometric effect of a mitochondrial crista electrostatically enhancing localized proton density to the crista tip where the ATP synthase can readily utilize the localized proton density to drive ATP synthesis. This represents another type of quantitative (optional) improvement that may be evolutionally advantageous, but not necessarily a universal feature to all organisms. This again can explain why seem only mitochondria have cristae formed so extensively; while some bacteria have an intracytoplasmic membrane structure similar to cristae but others may or may not have it^65^.

## Conclusion

This study employing the Lee transmembrane electrostatic proton localization theory^27–31^ has now shown that the localized proton associated pH ($-Lo{g}_{10}({[{H}_{L}^{+}]}_{tip}^{0})$) at a crista tip (or ridge) can be significantly lower (by as much as “─ 1.00” pH unit) than that ($-Lo{g}_{10}({[{H}_{L}^{+}]}_{flat}^{0})$) at the flat plane region within the same crista (Fig. 4, Eq. 18, and Table 2). Therefore, we can now understand, the formation of cristae with ATP synthase dimer rows located at the cristae ridges (tips) where the localized proton concentration (${[{H}_{L}^{+}]}_{tip}^{0}$) is significantly higher than that (${[{H}_{L}^{+}]}_{flat}^{0}$) at the relatively flat membrane plane regions where the redox-driven proton-pumping “respiratory supercomplexes” are situated is bioenergetically advantageous. That is, at the cristae ridges, the ATP synthase represents a sink for transmembrane electrostatically localized protons at the liquid-membrane interface, while the redox-driven proton pumps are in the relatively flat membrane plane regions of the cristae. Electrostatically guiding the localized protons to the ATP synthase at the cristae ridges, the liquid-membrane interface along the mitochondrial inner membrane surface may serve as a transmembrane electrostatically localized protonic conduction pathway (Fig. 4) for effective ATP synthesis without requiring any of the putative interfacial barrier^72^.

Furthermore, as shown in Fig. 2 and Table 1, the formation of cristae creates more mitochondrial inner membrane surface area and thus protonic capacitance, which enhances its ability to store transmembrane electrostatically localized charges (energy). For a typical mitochondrion with cristae at a membrane potential Δ*ψ* in a range from 50 to 200 mV, the numbers of transmembrane electrostatically localized protons per mitochondrion were calculated to be in a range from 1.84 × 10^4^ to 7.36 × 10^4^, which is 3 times more than that of a mitochondrion without cristae (Fig. 3). These results can now better explain the biological significance for mitochondrial cristae formation in relation to protonic bioenergetics.

Therefore, this study has now elucidated the pivotal architectural features of cristae membranes in relation to a curved protonic capacitor in accordance with the Lee transmembrane-electrostatic proton localization theory^27–31^: (1) the formation of mitochondrial cristae (Figs. 1 and 2) may be bioenergetically advantageous since it creates an extended membrane surface area not only for the accumulation of oxidative phosphorylation enzymes (respiratory chain complexes I to IV; F_1_F_o_-ATP synthase) but also for protonic capacitance to store electrostatically localized charges (energy); and (2) the shaping of mitochondrial cristae as a curved membrane micro-compartment (Fig. 1C and Fig. 4) may enhance protonic bioenergetics coupling for ATP production through the use of the proton-pumping “respiratory supercomplexes” (complexes I, III and V) at the relatively flat membrane plane regions and the ATP synthase enzymes at the cristae tips (ridges) where the transmembrane electrostatically localized proton concentration (${[{H}_{L}^{+}]}_{tip}^{0}$) can naturally be higher than that (${[{H}_{L}^{+}]}_{flat}^{0}$) at the flat membrane plane regions.

## Supplementary information

Supplementary information.

## Data Availability

All data generated or analyzed during this study are included in this article and the associated supporting information (xls efile showing the detailed calculations of the transmembrane electrostatically localized protons per mitochondrion) available online at the journal website.

## References

[1] Zick M, Rabl R, Reichert AS (2009). Cristae formation-linking ultrastructure and function of mitochondria. Bba-Mol Cell Res.

[2] Paumard, P. *et al*. The ATP synthase is involved in generating mitochondrial cristae morphology. *Embo J* **21**, 221-230, 10.1093/emboj/21.3.221 (2002). 10.1093/emboj/21.3.221 PMC125827 11823415

[3] Rabl R (2009). Formation of cristae and crista junctions in mitochondria depends on antagonism between Fcj1 and Su e/g. The Journal of cell biology.

[4] Paumard P (2002). Two ATP synthases can be linked through subunits i in the inner mitochondrial membrane of Saccharomyces cerevisiae. Biochemistry-Us.

[5] Giraud MF (2002). Is there a relationship between the supramolecular organization of the mitochondrial ATP synthase and the formation of cristae?. Bba-Bioenergetics.

[6] Vogel F, Bornhovd C, Neupert W, Reichert AS (2006). Dynamic subcompartmentalization of the mitochondrial inner membrane. J Cell Biol.

[7] Bornhovd C, Vogel F, Neupert W, Reichert AS (2006). Mitochondrial membrane potential is dependent on the oligomeric state of F1F0-ATP synthase supracomplexes. J Biol Chem.

[8] Jimenez L (2014). synthases cluster as discrete domains that reorganize with the cellular demand for oxidative phosphorylation. J Cell Sci.

[9] Colina-Tenorio, L., Horten, P., Pfanner, N. & Rampelt, H. Shaping the mitochondrial inner membrane in health and disease. *J Intern Med***n/a**, 10.1111/joim.13031 (2020). 10.1111/joim.13031 32012363

[10] Kuhlbrandt, W. Structure and function of mitochondrial membrane protein complexes. *Bmc Biol* **13**, 10.1186/s12915-015-0201-x (2015). 10.1186/s12915-015-0201-x PMC4625866 26515107

[11] Davies KM (2011). Macromolecular organization of ATP synthase and complex I in whole mitochondria. P Natl Acad Sci USA.

[12] Davies KM, Anselmi C, Wittig I, Faraldo-Gomez JD, Kuhlbrandt W (2012). Structure of the yeast F1Fo-ATP synthase dimer and its role in shaping the mitochondrial cristae. P Natl Acad Sci USA.

[13] Blum TB, Hahn A, Meier T, Davies KM, Kuhlbrandt W (2019). Dimers of mitochondrial ATP synthase induce membrane curvature and self-assemble into rows. P Natl Acad Sci USA.

[14] Wollweber F, von der Malsburg K, van der Laan M (2017). Mitochondrial contact site and cristae organizing system: A central player in membrane shaping and crosstalk. Bba-Mol Cell Res.

[15] Allegretti M (2015). Horizontal membrane-intrinsic alpha-helices in the stator a-subunit of an F-type ATP synthase. Nature.

[16] Davies, K. M. *et al*. Visualization of ATP Synthase Dimers in Mitochondria by Electron Cryo-tomography. *Jove-J Vis Exp*, 10.3791/51228 (2014). 10.3791/51228 PMC4828066 25285856

[17] Daum B, Walter A, Horst A, Osiewacz HD, Kuhlbrandt W (2013). Age-dependent dissociation of ATP synthase dimers and loss of inner-membrane cristae in mitochondria. P Natl Acad Sci USA.

[18] He JY (2018). Assembly of the membrane domain of ATP synthase in human mitochondria. P Natl Acad Sci USA.

[19] Althoff T, Mills DJ, Popot JL, Kuhlbrandt W (2011). Arrangement of electron transport chain components in bovine mitochondrial supercomplex I1III2IV1. Embo J.

[20] Davies KM, Blum TB, Kuhlbrandt W (2018). Conserved *in situ* arrangement of complex I and III2 in mitochondrial respiratory chain supercomplexes of mammals. yeast, and plants. P Natl Acad Sci USA.

[21] Guo RY, Gu JK, Zong S, Wu M, Yang MJ (2018). Structure and mechanism of mitochondrial electron transport chain. Biomed J.

[22] Cogliati S (2013). Mitochondrial Cristae Shape Determines Respiratory Chain Supercomplexes Assembly and Respiratory Efficiency. Cell.

[23] Murphy BJ (2019). Rotary substates of mitochondrial ATP synthase reveal the basis of flexible F-1-F-o coupling. Science.

[24] Hahn A (2016). Structure of a Complete ATP Synthase Dimer Reveals the Molecular Basis of Inner Mitochondrial Membrane Morphology. Mol Cell.

[25] Baker LA, Watt IN, Runswick MJ, Walker JE, Rubinstein JL (2012). Arrangement of subunits in intact mammalian mitochondrial ATP synthase determined by cryo-EM. P Natl Acad Sci USA.

[26] Palade GE (1953). An Electron Microscope Study of the Mitochondrial Structure. J Histochem Cytochem.

[27] Lee J (2012). Proton-electrostatics hypothesis for localized proton coupling bioenergetics. Bioenergetics.

[28] Lee J (2015). Proton-electrostatic localization: explaining the bioenergetic conundrum in alkalophilic bacteria. Bioenergetics.

[29] Lee JW (2019). Electrostatically localized proton bioenergetics: better understanding membrane potential. Heliyon.

[30] Lee JW (2005). A possible electrostatic interpretation for proton localization and delocalization in chloroplast bioenergetics system. Biophysical Journal.

[31] Lee, J. W. Localized excess protons and methods of making and using same. USA patent No. US 10,501,854 B2 (2019).

[32] Xiong JW, Zhu LP, Jiao XM, Liu SS (2010). Evidence for Delta pH surface component (Delta pH(S)) of proton motive force in ATP synthesis of mitochondria. Bba-Gen Subjects.

[33] Rieger, B., Junge, W. & Busch, K. B. Lateral pH gradient between OXPHOS complex IV and F0F1 ATP-synthase in folded mitochondrial membranes. *Nat Commun* **5**, 10.1038/ncomms4103 (2014). 10.1038/ncomms4103 24476986

[34] Saeed HA, Lee JW (2015). Experimental Demonstration of Localized Excess Protons at a Water-Membrane Interface. Bioenergetics.

[35] Chiang, G. G. & Dilley, R. A. Intact Chloroplasts Show Ca-2+-Gated Switching between Localized and Delocalized Proton Gradient Energy Coupling (Atp Formation). *Plant Physiol* **90**, 1513–1523, 10.1104/pp.90.4.1513 (1989). 10.1104/pp.90.4.1513 PMC1061919 16666959

[36] Weichselbaum, E. *et al*. Origin of proton affinity to membrane/water interfaces. *Sci Rep-Uk* **7**, 10.1038/s41598-017-04675-9 (2017). 10.1038/s41598-017-04675-9 PMC5495794 28674402

[37] Zhang C (2012). Water at hydrophobic interfaces delays proton surface-to-bulk transfer and provides a pathway for lateral proton diffusion. Proceedings of the National Academy of Sciences.

[38] Morelli, A. M., Ravera, S., Calzia, D. & Panfoli, I. An update of the chemv10.1098/rsob.180221 (2019). 10.1098/rsob.180221 PMC6501646 30966998

[39] Saeed H, Lee J (2018). Experimental determination of proton-cation exchange equilibrium constants at water-membrane interface fundamental to bioenergetics. WATER Journal: Multidisciplinary Research Journal.

[40] Lee, J. Localized excess protons and methods of making and using the same, PCT International Patent Application Publication No. WO 2017/007762 A1., 56 pages (2017).

[41] Guffanti A, Krulwich T (1984). Bioenergetic problems of alkalophilic bacteria. Biochem Soc T.

[42] Krulwich TA, Gilmour R, Hicks DB, Guffanti AA, Ito M (1998). Energetics of alkaliphilic Bacillus species: physiology and molecules. Advances in microbial physiology.

[43] Krulwich, T. A. *et al*. Adaptive mechanisms of extreme alkaliphiles. *Extremophiles handbook*, 119-139 (2011).

[44] Meier T (2007). A tridecameric c ring of the adenosine triphosphate (ATP) synthase from the thermoalkaliphilic Bacillus sp strain TA2.A1 facilitates ATP synthesis at low electrochemical proton potential. Mol Microbiol.

[45] Krulwich TA (1986). Bioenergetics of alkalophilic bacteria. The Journal of membrane biology.

[46] Krulwich TA, Guffanti AA (1989). Alkalophilic bacteria. Annual Reviews in Microbiology.

[47] Olsson K, Keis S, Morgan HW, Dimroth P, Cook GM (2003). Bioenergetic properties of the thermoalkaliphilic Bacillus sp. strain TA2. A1. J Bacteriol.

[48] McLaughlin S (1989). The Electrostatic Properties of Membranes. Annual Review of Biophysics and Biophysical Chemistry.

[49] Grahame DC (1947). The electrical double layer and the theory of electrocapillarity. Chemical reviews.

[50] Krulwich, T. A., Hicks, D. B., Swartz, T. & Ito, M. in *Physiology and Biochemistry of Extremophiles* (American Society of Microbiology (2007).

[51] Haiens TH, Dencher NA (2002). Cardiolipin: a proton trap for oxidative phosphorylation. Febs Lett.

[52] Liu J (2014). Cardiolipin Is Dispensable for Oxidative Phosphorylation and Non-fermentative Growth of Alkaliphilic Bacillus pseudofirmus OF4. J Biol Chem.

[53] Lee JW (2013). Membrane surface charges attracted protons are not relevant to proton motive force. Bioenergetics.

[54] Lee, J. Localized excess protons and methods of making and using the same. *United States Patent Application Publication No. US 20170009357 A1*, 73pp. (2017).

[55] Marx D, Tuckerman ME, Hutter J, Parrinello M (1999). The nature of the hydrated excess proton in water. Nature.

[56] Pomès R, Roux B (2002). Molecular mechanism of H+ conduction in the single-file water chain of the gramicidin channel. Biophysical journal.

[57] Marx D (2006). Proton transfer 200 years after von Grotthuss: Insights from ab initio simulations. Chemphyschem.

[58] Ohanian, H. C. in *Physics* Ch. 24, 565–573 (W. W. Norton & Company (1985).

[59] Saeed, H. Bioenergetics: Experimental Demonstration of Excess Protons and Related Features. *PhD Thesis, Old Dominion University, Norfolk, VA 23529 USA* (2016).

[60] Nicholls, D. G. & Ferguson, S. J. in *Bioenergetics (Fourth Edition)* 27-51 (Academic Press (2013).

[61] Mitchell P, Moyle J (1969). Estimation of Membrane Potential and pH Difference across the Cristae Membrane of Rat Liver Mitochondria. Eur J Biochem.

[62] Chinopoulos C (2009). A Novel Kinetic Assay of Mitochondrial ATP-ADP Exchange Rate Mediated by the ANT. Biophysical Journal.

[63] Mannella CA, Lederer WJ, Jafri MS (2013). The connection between inner membrane topology and mitochondrial function. J Mol Cell Cardiol.

[64] Zheng Y, Shojaei-Baghini E, Wang C, Sun Y (2013). Microfluidic characterization of specific membrane capacitance and cytoplasm conductivity of single cells. Biosens Bioelectron.

[65] Pinevich AV (1997). Intracytoplasmic membrane structures in bacteria. Endocytobiosis Cell.

[66] Kopek BG, Shtengel G, Xu CS, Clayton DA, Hess HF (2012). Correlative 3D superresolution fluorescence and electron microscopy reveal the relationship of mitochondrial nucleoids to membranes. P Natl Acad Sci USA.

[67] Dlaskova A (2019). Mitochondrial cristae narrowing upon higher 2-oxoglutarate load. Bba-Bioenergetics.

[68] Stephan, T., Roesch, A., Riedel, D. & Jakobs, S. Live-cell STED nanoscopy of mitochondrial cristae. *Sci Rep-Uk* **9**, 10.1038/s41598-019-48838-2 (2019). 10.1038/s41598-019-48838-2 PMC6712041 31455826

[69] Gu JK (2019). Cryo-EM structure of the mammalian ATP synthase tetramer bound with inhibitory protein IF1. Science.

[70] McDonald, K. T. Conducting Ellipsoid and Circular Disk. http://physics.princeton.edu/~mcdonald/examples/ellipsoid.pdf, 1-6 (2002).

[71] Strauss M, Hofhaus G, Schroeder RR, Kuhlbrandt W (2008). Dimer ribbons of ATP synthase shape the inner mitochondrial membrane. Embo J.

[72] Cherepanov DA, Feniouk BA, Junge W, Mulkidjanian AY (2003). Low dielectric permittivity of water at the membrane interface: Effect on the energy coupling mechanism in biological membranes. Biophysical Journal.

[73] Mulkidjanian AY, Heberle J, Cherepanov DA (2006). Protons @ interfaces: Implications for biological energy conversion. Bba-Bioenergetics.

